# Immunohistochemical evaluation of epithelial ovarian carcinomas identifies three different expression patterns of the MX35 antigen, NaPi2b

**DOI:** 10.1186/s12885-017-3289-2

**Published:** 2017-05-02

**Authors:** Kristina Levan, Matin Mehryar, Constantina Mateoiu, Per Albertsson, Tom Bäck, Karin Sundfeldt

**Affiliations:** 1 Sahlgrenska Cancer Center, Department of Obstetrics and Gynecology, Institute of Clinical Sciences, University of Gothenburg, SE-405 30 Gothenburg, Sweden; 2 Department of Pathology and Cytology, Institute of Biomedicine, University of Gothenburg, SE-405 30 Gothenburg, Sweden; 3 Department of Oncology, Institute of Clinical Sciences, University of Gothenburg, SE-405 30 Gothenburg, Sweden; 4 Department of Radiation Physics, Institute of Clinical Sciences, University of Gothenburg, SE-405 30 Gothenburg, Sweden; 5 Sahlgrenska Cancer Center, Department of Obstetrics and Gynecology, Institute of Clinical Sciences, University of Gothenburg, S-413 45 Gothenburg, Sweden

**Keywords:** Ovarian cancer, NaPi2b expression, Monoclonal antibody, Radiotherapy

## Abstract

**Background:**

To characterize the expression of the membrane transporter NaPi2b and antigen targeted by the MX35 antibody in ovarian tumor samples. The current interest to develop monoclonal antibody based therapy of ovarian cancer by targeting NaPi2b emphasizes the need for detailed knowledge and characterization of the expression pattern of this protein. For the majority of patients with ovarian carcinoma the risk of being diagnosed in late stages with extensive loco-regional spread disease is substantial, which stresses the need to develop improved therapeutic agents.

**Methods:**

The gene and protein expression of SLC34A2/NaPi2b were analyzed in ovarian carcinoma tissues by QPCR (*n* = 73) and immunohistochemistry (*n* = 136). The expression levels and antigen localization were established and compared to the tumor characteristics and clinical data.

**Results:**

Positive staining for the target protein, NaPi2b was detected for 93% of the malignant samples, and we identified three separate distribution patterns of the antigen within the tumors, based on the localization of NaPi2b. There were differences in the staining intensity as well as the distribution pattern when comparing the tumor grade and histology, the mucinous tumors presented a significantly lower expression of both the targeted protein and its related gene.

**Conclusion:**

Our study identified differences regarding the level of the antigen expression between tumor grade and histology. We have identified differences in the antigen localization between borderline tumors, type 1 and type 2 tumors, and suggest that a pathological evaluation of NaPi2b in the tumors would be helpful in order to know which patients that would benefit from this targeted therapy.

## Background

MX35 is a monoclonal antibody targeting the sodium-dependent phosphate transport protein 2B (NaPi2b) gene name *SLC34A2*. The normal expression is in epithelial cells like type II pneumocytes, brush border membrane of small intestine and in the mammary gland [1, 2]. The protein is involved in actively transporting phosphate ions into the cell by a Na^+^ co-transport [3–7]. Protein expression is further evident in female genital tract, endometrium, cervix and fallopian tube [8]. While normal ovary has been reported to lack expression of NaPi2b the expression is high in epithelial ovarian cancer (EOC) NaPi2b is expressed in 80–100% of the tumors [3, 5, 6, 9, 10]. EOC is the most prevalent type of ovarian cancer (90%), and consists of five pathological subtypes: serous, mucinous, clear cell, endometrioid and undifferentiated carcinoma [11]. Standard treatment includes optimal debulcing surgery followed by first-line chemotherapy in selected cases.

Current interest in targeting the NaPi2b protein in ovarian cancer by use of monoclonal antibodies either conjugated to alpha-emitting radionuclides [12–14], or as antibody drug conjugates [15] has highlighted the importance of evaluating the antigene expression in tumor samples. In the situation of using alpha emitting radionuclides i.e. targeted alpha therapy (TAT) for ovarian cancer, recently also explored for other antigenic targets than NaPi2b, a solution containing antibodies labeled with α-particles emitting radionuclide is injected locally into the peritoneal cavity [16]. The short ranged α-particles (<0.1 mm) used in TAT make them especially suitable to eradicate minimal residual disease, since a large portion of the radiation energy can be confined to the cancer cells only. At the same time, due to the short range a too large heterogeneity of the intratumoral distribution and/ or intensity of the antigen could impact the therapeutic outcome. This has been shown on epithelial ovarian cancer (EOC) biopsies where the tumor uptake (%ID/g) radiolabeled MX35 could vary a factor of 20 in-between samples and that the activity uptake of MX35 correlated both with level and intensity of the MX35-antigen expression, as analyzed by autoradiography and immunohistochemistry [17]. Bioimaging of metastases in animal models of ovarian cancer has shown heterogenic distribution on small tumors of varying sizes [18, 19]. Strategies to predict and counteract for the impact of heterogeneity are currently being investigated, including parameters like radiation crossfire and specific activity of the radiopharmaceutical [20].

Nevertheless, detailed information about the antigen expression pattern within the tumor mass, is crucial for small scale dose calculation and prediction of the biological outcome of the radiotherapy. Therefore, knowledge about the actual expression pattern of NaPi2b in different histologies, grades and stages of ovarian tumors (OT) is warranted.

In this report we analyzed the localization and expression pattern of the NaPi2b protein (*n* = 136) as well as its gene expression (*n* = 73) (SLC34A2) in fresh frozen ovarian borderline and malignant tumor samples. The results are described and correlated to clinical pathology. The number of samples included in previous expression pattern studies of NaPi2b in ovarian cancer range from *n* = 14–50 [4, 7, 9, 21], and our objective was to establish the antigen expression in a larger set of EOC samples.

## Methods

### Tumor samples

Ovarian tumor tissues were subjected to analysis by quantitative polymerase chain reaction (QPCR) (*n* = 73, benign *n* = 5, borderline *n* = 11, malignant *n* = 57) and immunohistochemistry (IHC) (*n* = 150, malignant *n* = 108, borderline *n* = 42) (histology, stage and grade are described in Table 1.). The tumor samples were collected prospectively and consecutively from patients diagnosed from March 2001 to September 2010 with suspected cystic pelvic tumor as part of another study [22]. Ovarian biopsies from 14 women without ovarian cancer were used as control tissue. The local ethical committee at the University of Gothenburg approved the study, and each patient gave her informed, written consent. All case diagnoses were reviewed by a gynecological pathologist using established morphologic criteria according to World health organization (WHO) 2003 [23]. Fresh frozen biopsies from each tumor were divided into two samples one was used for RNA extraction and the other one was paraffin embedded and used in the tissue micro array (TMA). The staining intensity and pattern were evaluated according to histology and the dualistic model presented by Shih et al. [24] Type I included low-grade (G1) serous, low-grade (G1) endometrioid, all clear cell, and mucinous carcinomas. Type II included high-grade (G2–G3) serous, high-grade (G2–G3) endometrioid, undifferentiated carcinoma, and malignant mixed mesodermal tumors [25].

**Table 1:** Malignant and borderline samples included in the analysis

				Stage					Grade			
	Total	Borderline	Malignant	I	II	III	IV	N/A	Highly	Moderately	Poorly	Undiff.
Serous	90	22	68	36	8	39	5	2	16	17	32	3
Mucinous	29	19	10	25	1	1	1	1	6	2	1	1
Clear cell	8	-	8	6	-	2	-	-	5	2	1	-
Endometrioid	16	1	15	10	1	5	-	-	5	5	5	-
Undifferentiated	7	-	7	3	-	4	-	-	-	-	2	5
total	150	42	108	80	10	51	6	3	32	26	41	9

Presented based on histology, stage and grade respectively

### Quantitative polymerase chain reaction (qPCR)

RNA extraction was performed using QIAGEN RNeasy plus Mini Kit (QIAGEN, Germany) according to the manufacturer’s manual, and the RNA concentration was measured with the NanoDrop instrument (ND1000 software, Thermo Fisher Scientific, Wilmington, DE) (Table 1). The RT-PCR High-Capacity cDNA Reverse Transcription kit (Applied Biosystems, Foster City, CA) was used to produce cDNA from the RNA samples. TaqMan Universal PCR Master Mix (Applied Biosystems, Focter City, CA), probe and primers for *SLC34A2* (Hs 00197519_m1) as the target gene and *GUSB* (Hs 99999908_m1) as the reference gene (Life Technologies Corporation, San Diego, CA) were used. A 7000 sequence detection system (Applied Biosystems, Foster City, CA) was used to determine the expression levels by QPCR of the target gene for all samples. Pooled normal ovarian tissue (*n* = 7) was used as control since it was previously reported to contain low levels of *SLC34A2* [2, 5, 21, 26, 27]. The Ct values were used to calculate ΔΔCt and fold change (FC) for each tumor sample.

### MX35 antibody

MX35 is a murine IgG1 monoclonal antibody specifically directed towards a membrane phosphate transporter protein (NaPi2b). The murine MX35 antibody was produced from a hybridoma line and was kindly provided by The Ludwig Institute for Cancer Research (New York, NY, USA). The hybridoma cells were cultured at the Department of Cell and Molecular Biology at the University of Gothenburg (Gothenburg, Sweden) and the antibody was purified from hybridoma supernatant by protein-A chromatography at the Department of Radiation Physics at the University of Gothenburg (Gothenburg, Sweden) [28].

### Immunohistochemistry (IHC)

For the TMA, the whole biopsy was sectioned and stained with Hematoxylin (Histolab Products AB, Sweden). Three representative tumor areas were identified under the light microscope (Olympus BX45, Olympus Corporation, Tokyo, Japan), and three cores of 1,0 mm-diameter were punched with a manual tissue microarrayer (Beecher MTA-1, Estigen,Tartu, Estonia) and re-embedded into a predefined position on a new, empty, paraffin block. The TMA block was heated at 45 °C in 1 h, sectioned, 4 μm, and mounted onto slides.

For IHC analysis, the TMA slides were immunostained by UltraVision Quanto Detection System HRP DAB kit (Thermo Fisher Scientific, Wilmington, DE) and incubated overnight with the MX35 antibody at a concentration of 1:1000. All slides were counterstained with hematoxylin and mounted with Pertex (Histolab Products AB, Sweden). All TMAs were scanned by a Leica SCN400 (Leica Microsystems, Milton Keynes, UK). SlidePath Gateway LAN software was used for the evaluation of the NaPi2b distribution.

### NaPi2b expression

Staining for NaPi2b were estimated for each tumor and the amount of positive cells were evaluated and given a value; no cells stained = 0, 1/3 = 1, 1/3 > 2/3 = 2, >2/3 = 3. The intensity was estimated for each tumor no staining (negative) = 0, light yellow to yellow (weak) = 1+, light brown (moderate) = 2+, and dark brown (strong) = 3+. For the correlation analysis between QPCR and IHC we used a scoring system were we combined the intensity with the amount of cells stained in the tumor sample described by Tomic et al. [29]. The amount of cells stained (0–3) was used together with the intensity, to calculate a score that describes a combination of both the intensity and the amount of stained cell for each tumor. The product (amount of cells stained multiplied with intensity) ranging from zero to nine were grouped into four final scores as follows: score 0, score 1 (low 1–3), score 2 (intermediate 4–6) score 3 (high >7) [29].

### Statistics

The differences in expression of *SLC34A2* between the groups previously described, were evaluated using unpaired two-sample Student’s t-test (IBM® SPSS® Statistics) and were considered significant if *P* < 0.05. In the analysis all samples were compared to the normal ovarian expression level, which was set to one. Correlation between the gene and protein expression was calculated using Pearson correlation. ANOVA test was used to analyze the variance of NaPi2b expression between the groups. Box plots of tumors grouped into stage, grade and histology were drawn to illustrate the ANOVA analysis results. Two researchers (MC and KL) independently evaluated the IHC staining of the TMAs. In order to evaluate their inter-rater agreement Cohen’s kappa coefficient was calculated.

## Results

### SLC34A2 gene expression analysis

**Fig. 1:**
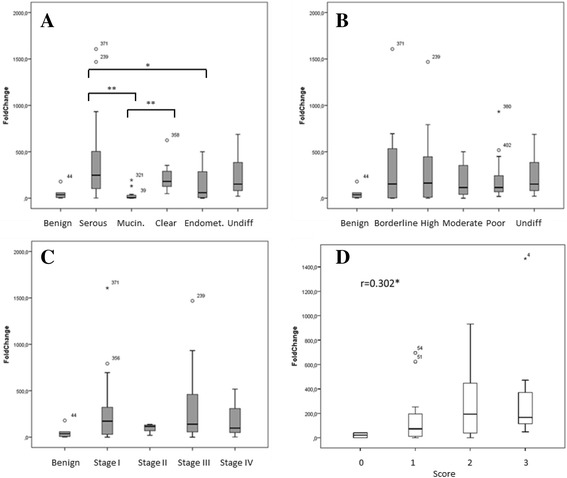
Boxplots illustrating the level of SLC34A gene expression. **a**) level of expression in relation to the different histologies, with significant differences in expression between mucinous and both clear cell and serous (**P < 0.01), and significant differences between endometrioid and serous tumors (*P < 0.05,); **b**) in relation to grade and in **c**) to stage. **d**) Boxplot illustrates the correlation between the staining of NaPi2b and the gene expression of SLC34A, Pearson correlation *r* = 0.302*

To compare the expression levels of the *SLC34A2* gene, coding for NaPi2b, among the different classifications of the ovarian tumors (OT), we subdivided the samples into groups based on histology, grade and stage (Table 1). The gene expression analysis of *SLC34A2* displayed considerable variation in expression levels of this gene within the material, with values ranging from no expression up to a FC > 1600 (mean = 237; median = 126) compared to the expression in normal ovaries. The mucinous OT demonstrated a significantly lower expression of *SLC34A2* than both the serous and the clear cell OT (*P* = 0.007 and *P* = 0.002 respectively) (Fig. 1a). We found no significant difference between mucinous and endometrioid OT (*P* = 0.062). Endometrioid OT had significantly lower expression levels than the serous OT (*P* = 0.038) (Fig. 1a).

### MX35 staining of NaPi2b

For evaluation of the NaPi2b expression in the tumors, six TMAs containing a total of 150 ovarian OT samples were stained with the MX35 antibody and scanned for analysis, 108 malignant and 42 borderline tumors (Table 1). Of the 150 OT 14 samples (9%) were excluded from the analysis (ten malignant and four borderline) due to lack of tumor cells in the TMA. For the remaining 136 samples, quantification of the staining intensity and localization of NaPi2 was performed. For interrater reliability, Cohen’s kappa coefficient was calculated for intensity (κ =0.77) and for pattern (κ =0.89), which established a robust IHC assessment. We found that 127 (93%) out of the 136 samples were positively stained. A Pearson correlation analysis between the gene and protein expression in the tumor tissues was performed and a positive correlation was established (*r* = 0.302, *P* < 0.05) (Fig. 1d).

**Fig. 2:**
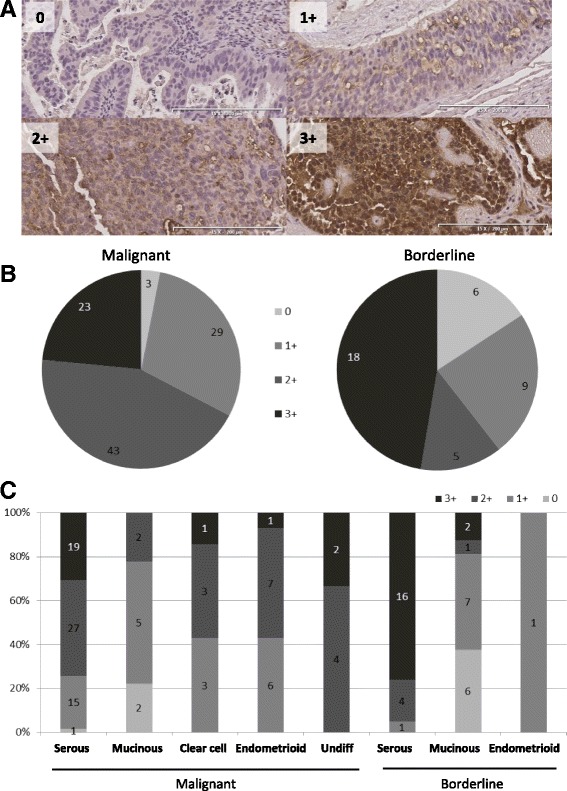
Illustrative images of the staining intensities and the distribution of the different intensities among the samples. **a**) Representative images demonstrating the different staining intensities Upper left: no staining = 0, serous (highly differentiated stage I), Upper right: 1 = weak staining (endometrioid poorly differentiated, stage II). Lower left: 2 = moderate staining (endometrioid poorly differentiated stage III. Lower right: 3 = strong staining, serous poorly differentiated stage III). **b**) Malignant and borderline tumor samples divided in scored staining intensity. **c**) Bars illustrating the samples divided into histology and how the staining intensities were distributed within their histological group

Among the 136 samples there were 41 tumors (30%) stained at the highest level (3+), 48 tumors (36%) as 2+, 38 tumors (28%) as 1+ and nine tumors (7%) did not show any staining at all (Fig. 2a-b). Six of the negatively stained tumors were mucinous borderline tumors. The three malignant tumors with no staining were all type 1, two were mucinous adenocarcinoma, one highly and one moderately differentiated, and one was highly differentiated serous adenocarcinoma. The borderline tumors had a higher number of cases with 3+ staining compared to the malignant tumors, 47% and 29% respectively.

**Fig. 3:**
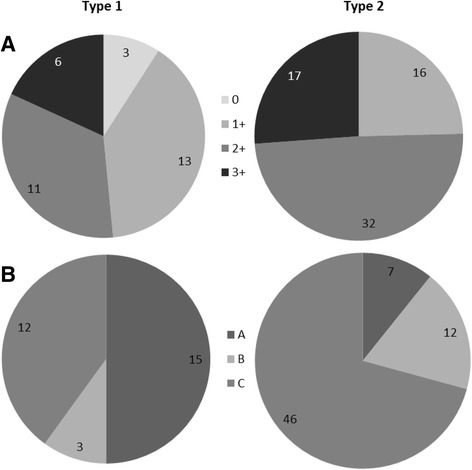
Results presented in relation to tumor type 1 and 2. **a**) Upper panel present the tumors according to type and intensity of staining, **b**) The tumors are grouped according to type and presented by the pattern they present

We subdivided the material according to histology, grade and stage. When comparing the histologies we were able to identify differences in the staining intensity between the groups (Fig. 2b). The serous tumors showed highest number of tumors with 3+ staining between the histologies, and among the mucinous tumors only two out of 29 were considered to be 3+ both of them were borderline tumors (Fig. 2c). The majority of the mucinous samples had negative or 1+ staining in both the malignant (75%) and in the borderline tumors (81%). The low expression of the target protein NaPi2b in the mucinous tumors correlates well with the low gene expression of *SLC34A2* in this group. The malignant tumors were grouped according to type (type 1: *n* = 33, type 2: *n* = 65) [22, 24], among the type 1 tumors 48% (*n* = 16) had 1+ or no staining, compared to only 25% with 1+ tumors in the more aggressive type 2. Further, there were 75% of the tumors that were considered as 2+ or 3+ in the type 2 tumors (Fig. 3).

### Distribution pattern of NaPi2b

**Fig. 4:**
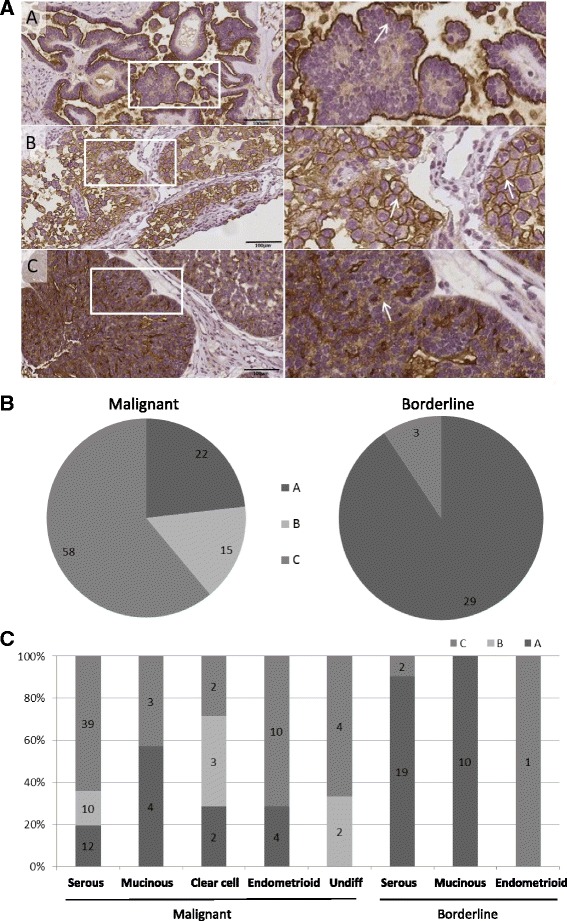
Illustration presenting the three indicated staining patterns and their distribution among the samples. **a**) Illustration of the three characteristic MX35 staining patterns identified among the samples; Pattern **a**, the target was located in the cell membranes of the cells close to the surface of the tumor (serous borderline, stage I). Pattern **b**, MX35 staining in all of the tumor cell membranes over the entire tumor (clear cell highly differentiated, stage I). Pattern **c**, includes the tumors with a mixed staining pattern including staining of membranes in addition to cytoplasmic staining spreading inside the as well (serous poorly differentiated stage I). The right panel shows images with higher magnification to give a more detailed view of the three patterns. **b**) The distribution of the tumor samples, malignant and borderline, between the three patterns. **c**) The tumors divided based on pattern, presented according to their histology

Because of the protein function and results from previous studies it was expected to find NaPi2b located to cell membranes [3, 4, 6, 7]. When we evaluated the TMAs we identified differences in the staining pattern between the tumors (Fig. 4). We identified three different patterns for the distribution of NaPi2b (Fig. 4). In pattern A) NaPi2b was primarily located in the cellmembranes of cells close to the surface of the tumor. Even when the tumor consisted of several layers of epithelial cells staining was only detected for epithelial cells that were located in the external layer of the tumor (Fig. 4a). In pattern B) NaPi2b was not limited to the cell membranes in the cells at the surface of the tumor but was found in all of the tumor cell membranes. Finally, in pattern C) there was a mixed staining pattern with NaPi2b localized to both the cellmembranes and the cytoplasm of the same cell (Fig. 4c). Staining of normal ovarian tissue (*n* = 4 women) showed absence of MX35 in follicles, stroma and ovarian surface epithelium in ¾, and one had typical pattern A staining of ovarian surface epithelium only. Two of 3 women with endometriosis, originating from the uterus, had pattern A staining (data not shown). Of the 136 tumors, borderline and malignant, we were able to subdivide 126 samples into three groups according to the staining pattern (A, B and C). The majority of borderline tumors (*n* = 29, 90%) had pattern A, and only three borderline tumors had both membrane and cytoplasmic, pattern C and none showed pattern B (Fig. 4d). Conversely, the majority of samples among the malignant tumors displayed pattern C (*n* = 57, 61%) (Fig. 4d). Both pattern A and B were represented among these tumors, 23% and 16% respectively. With regard to histology, pattern C was most common in the serous (64%), endometrioid (71%) and the undifferentiated OT (67%). Pattern A was the most common in mucinous OT (67%) (Fig. 4e). All three patterns were represented in the seven clear cell OC (A *n* = 2, B *n* = 3, C *n* = 2). None of the mucinous and endometrioid tumors displayed pattern B (Fig. 4e). In type 1 OT pattern A was present in 50% (*n* = 13) of the cases, contrary to type 2 OT were the most frequent was pattern C which was detected in 71% (*n* = 46).

## Discussion

The main objective of this study was to characterize the expression of the NaPi2b in ovarian tumor samples. The development, evaluation and optimization of the targeted antibody treatment for this patient group call for a more detailed characterization of the cancer cell antigen expression. Our results from this study complement the present knowledge of NaPi2b expression in epithelial ovarian cancer and ovarian borderline tumors.

With 93% of the ovarian cancer tissue samples positively stained our data shows a higher frequency of NaPi2b expression compared to a study performed by Lopes dos Santos et al. where 80% of the ovarian cancers express the protein [10]. Among the samples positive for NaPi2b the samples were evenly distributed between the staining intensities. All of the type 2 tumors were positive for NaPi2b and three out of four tumors had moderate or strong staining. Strong staining was more frequent among the serous borderline tumors, which is promising if targeted antibody based radiotherapy will be used in this group of patients. On the other hand, the majority of the mucinous tumors, both malignant and borderline, had low or negative staining compared to the serous tumors suggesting that theses tumors are not the ones that would benefit from this therapy. MX35 is designed to target NaPi2b expressed in the ovarian tumor cells and the antibody is suitable to carry a radionuclide that can deliver its energy to the target cells. It has been reported that other cancer types such as lung cancer, renal cancer and thyroid cancer also express the NaPi2b antigen, which suggests that this antibody may be useful in treatment of other cancer.

The MX35 staining patterns varied between the tumors, introducing novel information on how the antigen is distributed in the tissue. We classified the tumors according to three different staining patterns, and we believe that these differences in localization of the antigen are important factors governing the uptake and efficiency of a potential therapy targeting this protein. In the database, Human Protein Atlas, the pattern A, staining of the apical membrane, facing the surface of the tumor, was the dominating form represented in the samples shown for different types of tissue, including normal fallopian tubes, uterus and lung. For the normal tonsil there was an example with staining only in the cytoplasm, which would represent a forth pattern which is not represented in this study [30]. We found that normal ovaries were mostly without staining. If present, like in endometriosis lesion, pattern A was noticed, which is well in line with previous data [2, 5, 21, 26, 27]. In samples taken from cancer tumors presented at the human protein atlas database the most common pattern was A, but there were a few samples that showed some cytoplasmic staining in addition to staining at the surface of the cells [30]. In contrast to the findings of Shyian et al., who describe staining of NaPi2b predominantly at the surface membrane of cancer cells in well differentiated serous and endometrioid ovarian cancer [21], we identified pattern C (both membrane and cytoplasm) as the most common pattern for the malignant tumors, represented in 61% of the tumors. For borderline samples pattern A was presented in 90% of the cases and the remaining tumors showed pattern C. In concordance with Soares et al., we identified pattern B, staining in all the membrane of all layers of cancer cells, as the least common pattern with fifteen malignant tumors presenting pattern B [7].

There were differences in the expression levels of NaPi2b between histologies i.e. in clear cell carcinoma the levels of MX35 staining was of higher intensity than for the mucinous tumors (Fig. 2), this was in agreement with previous study by Soares and colleagues [7]. The expression of *SLC34A2* differed significantly among the histological groups with a less pronounced expression mainly in the mucinous tumors but also in the endometrioid tumors (Fig. 1). In contrast to previous studies we did not see any typical association between increased expression of SLC34A2 and differentiation grade of the tumors [5, 26]. On the contrary we were able to identify distinct differences when the NaPi2b staining was examined in relation to type, rather than differentiation grade. Type 2 tumors had higher staining intensity and presented more tumors with pattern C. High intensity staining in the cells could be a beneficial quality for the use of tailored immunotherapy strategies.

We detected low levels of the gene expressed in the benign samples, where the levels were within the same range as the mucinous malignant tumors, further emphasizes the importance of analyzing the expression of MX35 staining in biopsies from patients in order to ensure whether or not the patient could benefit from this type of therapy. In the majority of the samples the gene and protein expression correlated, but the inconsistencies between the gene and protein expression could be explained by the use of two separate tumor pieces, even though they were taken from the same tumor sample. In work with patient material it is important to acknowledge the heterogeneity of the tumors, both clinically and with respect to the tumor biology.

Ovarian cancer is characterized by unspecific symptoms and late diagnosis. At the time of diagnosis the majority of women have advanced stage disease with metastatic spread primarily in the abdominal cavity. It is therefore hypothesized that radio immunotherapy with the α-particle emitter ^211^At bound to a MX35 antibody has the potential to be beneficial for such patients. This type of targeted therapy may be used after primary staging and debulking surgery, which includes at least the removal of all visible tumor mass, both adnexa, the uterus and the omentum. Preclinical studies with ovarian cancer have established the efficiency and toxicity of this treatment supporting this notion [31, 32].

The interest in using α-particles in targeted therapy is increasing and TAT is being considered for many different cancers [14]. The TAT-regimen under current development, using MX35 with the α-particle emitter ^211^At, is a consolidating loco-regional therapy aimed to treat peritoneal microscopic disease in patients relapsing after surgery and chemotherapy [12]. The translation to clinical trial was made after very promising results in a series of preclinical studies [31–33]. With future developments, TAT therapy may be complemented with a systemic regimen aimed to treat vascularized and/or extra-peritoneal tumors. For this purpose, the MX35 antibody could possibly be used non-radiolabeled as suggested by data from a recent study of Rebmab200, a humanized version of MX35 [10]. In a recent study the murine MX35, used in the present study, was compared with its humanized counterpart (Rebmab200) and the antigen binding properties and in vivo behavior were found to be very similar [28]. Further, with the implementation of a pre-targeted radioimmunotherapy, a systemic TAT might be a possibility for the future. For these targeted strategies the anti-tumor efficacy will depend on the antigen expression and its intra-tumoral distribution i. e the targeted antigen [10, 34]. We have previously shown that, due to the short range of α-particles, the levels of absorbed radiation dose to the tumors, and other organs, could vary greatly depending on the distribution of radiolabeled antibody [18, 19].

There has been an increase in the use of antibodies within the field of targeted therapy [14, 35]. The antigen NaPi2b is currently being explored as a target for antibody based immunotherapy in ovarian and pulmonary cancer [10, 12, 36]. It is of fundamental importance to know the antigen expression frequency as well as the cellular localization of the antigen before treatment, this will be especially important for the targeted radiotherapies involving short-ranged α-particle irradiation, recently being explored for e.g. ovarian cancer [16, 37]. Our results suggest that there are differences regarding the level of the antigen expression between histologies and distinguish the mucinous tumors with a significantly lower expression of the antigen. Hence, a pathological evaluation of NaPi2 in the tumors that are surgically removed would give information on which patients that would benefit the most from a targeted therapy of this type. Furthermore, the presented data regarding the distribution of the NaPi2b antigen provide new knowledge for further development of antibody based therapy regimens of ovarian cancer.

## Conclusions

Our study identified differences in the level of the antigen expression and in the antigen localization between borderline tumors, type 1 and type 2 tumors, and we therefore suggest that a pathological evaluation of NaPi2b expression in the tumors would be helpful in order to know which patients that would benefit from a therapy targeting this antigen.

## Abbreviations

EOC: Epithelial ovarian carcinoma
IHC: Immuno histo chemistr
OT: Ovarian tumors
QPCR: Quantitative polymerase chain reaction
TAT: Targeted alpha therapy
TMA: Tissue microarray
WHO: World health organization
